# Comparison of Cholic Acid (MT921) and Deoxycholic Acid (DCA) in Fat Reduction Efficacy and Skin Adverse Reactions in Mini Pigs and Rodent Models

**DOI:** 10.3390/ph18111643

**Published:** 2025-10-30

**Authors:** Sujin Cho, Deu John M. Cruz, Minhee Shin, Jaeyoon Byun, Junho Lee, Seongsung Kwak

**Affiliations:** Medytox Gwanggyo R&D Center, 114 Central town-ro, Yeongtong-gu, Suwon-si 16506, Republic of Korea; sjcho@medytox.com (S.C.); deujohn.cruz@medytox.com (D.J.M.C.); minishin@medytox.com (M.S.); bjy2689@medytox.com (J.B.); jlee@medytox.com (J.L.)

**Keywords:** injection lipolysis, lipodissolve, cholic acid, deoxycholic acid, magnetic resonance imaging, subcutaneous fat, skin adverse reaction

## Abstract

**Background/Objectives:** This study compared fat reduction efficacy of cholic acid-based formulation (MT921) and deoxycholic acid (DCA), as well as skin adverse reactions (ADR), in mini pigs, mice, and rats. DCA is the active pharmaceutical ingredient found in several fat-dissolving injectables, such as Kybella^®^, V-OLET^®^, and Bellacholine^®^. **Methods:** In one study, single subcutaneous (s.c.) injections of 1.5% MT921 and 1% DCA were administered to the back of a mini pig at different sites and time points to ascertain histopathological events. In another study, three mini pigs received six repeated s.c. injections of 1.5% MT921 and 1% DCA at 4-week intervals, and changes in subcutaneous fat volume were monitored by magnetic resonance imaging (MRI), along with visual examination for ADRs. Additional ADRs were assessed in rodents, such as ulcerative dermatitis (UD) following MT921 and DCA s.c. injections in ICR/CD1 mice, and footpad edema after intraplantar injections in SD rats. **Results:** In mini pigs, 1.5% MT921 and 1% DCA induced comparable localized fat necrosis, accompanied by inflammatory cell influx and fibrosis. Also, repeated injections of 1.5% MT921 and 1% DCA induced comparable fat volume reduction in outer subcutaneous layer, though changes in middle subcutaneous layer was unaffected. Notably, MT921 evoked milder ADR based on lower incidence of hematoma and absence of nodules in mini pigs, less severe UD in mice, and reduced edema in rats. **Conclusions:** Local injections of 1.5% MT921 demonstrated fat-reduction efficacy comparable to 1% DCA while eliciting fewer and milder ADRs, supporting MT921 as a promising alternative lipolytic agent.

## 1. Introduction

Injection lipolysis is a minimally invasive procedure that removes unwanted fat from targeted areas by locally injecting a pharmacological agent to break down adipose tissue. This procedure was first introduced to the US in 2002 as a nonsurgical alternative to lipoplasty (liposuction) and used Lipostabil^®^ (Sanofi), an injectable that contained phosphatidylcholine (PC) combined with deoxycholate (DC) [1,2]. Since then, this treatment has been widely used in cosmetic surgery clinics and was generally called “lipodissolve”. Cosmetic surgery practitioners have used Lipostabil^®^ and other formulations that contain PC and DC, along with other ingredients, that promote fat removal to perform body-sculpting procedures on the abdomen, flanks, thighs, arms, back, and chin. Despite clinical evidence supporting the effectiveness of injection lipolysis, health authorities have strongly warned of the potential harm, as there is insufficient data on clinical safety and most, if not all of the formulations used, have not been approved for the intended purpose [3,4].

In 2015, deoxycholic acid (DCA) (development name: ATX-101, Kythera Biopharmaceuticals, Inc.), a secondary bile acid, became the first and only FDA-approved injection lipolysis drug intended for treatment of submental fat (double chin) [5,6]. It is currently manufactured by AbbVie/Allergan and marketed under the brand names Kybella^®^ (US) and Belkyra^®^ (Canada, EU, and Australia). Other injection lipolysis marketed brands that use DCA as active ingredient include Aqualyx^®^ (Marllor Biomedical), Bellacholine^®^ (LG Chem), and V-OLET^®^ (Daewoong Pharmaceuticals).

Despite its clinical efficacy, there are significant concerns regarding skin adverse reactions to DCA since its salt form (deoxycholate) is one of the more potent surfactants among bile salts. Preclinical studies have shown that DCA exerts nonspecific cytolytic effects not only on adipocytes, but also on surrounding tissues, such as muscle fibers, vascular endothelium, and peripheral nerves [7,8,9]. Clinical administration of DCA has been frequently associated with local adverse events, including pain, burning sensation, numbness, bruising, edema, pigmentation, and induration at the injection site [10,11]. In rare cases, marginal mandibular nerve injury has also been documented during submental fat treatment [12]. This highlights an unmet need for alternative agents with comparable lipolytic efficacy, but with better safety and tolerability profiles.

Recently, Medytox Inc. developed a new injection lipolysis drug using cholic acid-based formulation (development name: MT921, marketed name: NUVIJU^®^), a primary bile acid, for treatment of submental fat [13,14]. A randomized, double-blind, placebo-controlled, multi-center, Phase III study to evaluate the efficacy and safety of MT921 in subjects with moderate-to-severe submental fat was completed (Clinicaltrials.gov identifier: NCT05195112), and a New Drug Application (NDA) was approved by the Korean Ministry of Food and Drug Safety (MFDS) on 19 September 2025.

Bile acid salts act as amphiphilic surfactants that self-associate in aqueous media above their critical micellar concentration (CMC), forming micelles that can solubilize membrane lipids. Reported CMC values (assay- and condition-dependent) are approximately 7–8 mM for sodium cholate and 2–3 mM for sodium deoxycholate, indicating greater hydrophobicity and membrane-disruptive capacity of the latter [15,16,17]. The molar concentration of 1.5% cholic acid (≈37 mM) and 1% deoxycholic acid (≈25 mM) used in the present study is well above their respective CMC values, implying that micelle-driven lipid solubilization of the anionic (salt) forms of their respective formulation contributes to their lipolytic activity.

Here, we investigated the histological features, fat volume reduction, and skin adverse reactions (ADR) resulting from subcutaneous injection of MT921 in the mini pig model and compared them to those of DCA subcutaneous injections. For this purpose, 0.2 mL injections of 1.5% (*w*/*v*) MT921 and 1% (*w*/*v*) DCA were used to observe and compare the effects under the conditions applied in the clinical setting [6,13]. As a supplementary study, other ADR, such as ulcerative dermatitis and local edema, were also investigated using two rodent models.

## 2. Results

### 2.1. Histopathological Events Following MT921 and DCA Subcutaneous Injections in Mini Pig

**Figure 1:**
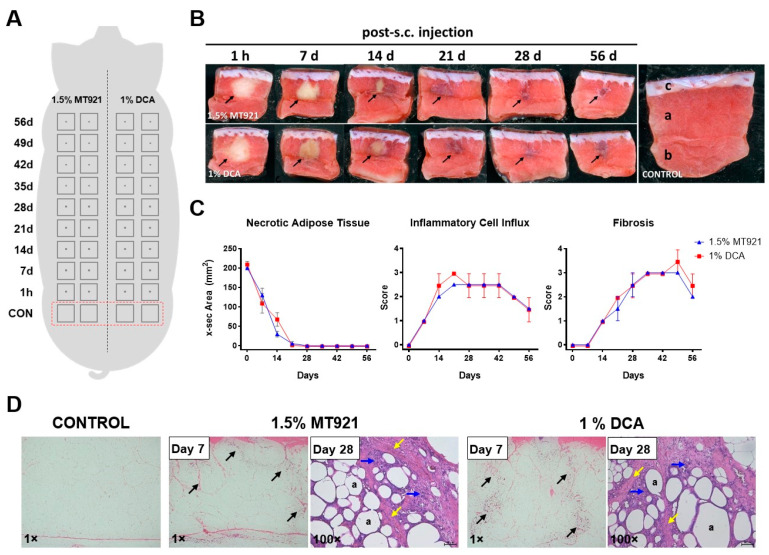
(**A**) Diagram illustrating injection sites for 1.5% MT921 and 1% DCA s.c. injections at the back of a mini pig at different time points and control sites (CON). (**B**) Representative TTC-stained tissue cross sections of MT921 (**top left**) and DCA (**bottom left**) injection sites that highlight major histopathological findings; arrows mark regions of necrotic fat (unstained) and fibrotic tissues (bluish-purple); control site (right) showing outer subcutaneous (a), middle subcutaneous (b), and dermis layers (c). (**C**) Cross-sectional area of fat necrosis and histologic scores for inflammatory cell influx and fibrosis (on a scale of 0–4) at each injection site representing different time points post-injection (*n* = 2); data points and error bars represent mean ± standard deviation; changes in cross-sectional area of necrotic tissue was analyzed using two-way ANOVA with Dunnett’s multiple comparisons test. (**D**) Representative histological specimens of control (non-treated), MT921, and DCA injection sites by hematoxylin and eosin staining that highlight major histopathological findings; necrotic tissue (black arrows), inflammatory cells (blue arrows), fibrotic tissue (yellow arrow), and adipose cells (a); magnification indicated on bottom left.

A reverse time-course approach to investigate the sequence of histopathological events was performed by injecting designated sites on the back of a mini pig with single subcutaneous (s.c.) injections of 1.5% MT921 and 1% DCA (0.2 mL, 5 mm depth) at the indicated times and then simultaneously 1 h after the last injection (Figure 1A). Tissue cross sections of the injection sites stained with TTC (triphenyltetrazolium chloride) revealed necrotic fat tissue (unstained region), with x-sec areas measuring 199.8 ± 0.9 mm^2^ and 210.9 ± 12.0 mm^2^, 1 h after s.c. injections of 1.5% MT921 and 1% DCA, respectively. At 2 weeks (Day 14), the necrotic tissue cross section area had decreased to 29.8 ± 10.3 mm^2^ and 69.6 ± 24.7 mm^2^, respectively, interspersed with fibrotic tissue (bluish-purple regions). By day 21, necrotic area at MT921 and DCA injection sites had diminished to 5.3 ± 7.5 mm^2^ and 3.2 ± 4.5 mm^2^, respectively (*p* < 0.05), and replaced with fibrotic tissues. From day 28, necrotic tissue was no longer visible, while fibrotic tissue remained visible at the injection sites up to day 56. These histopathological events were found to occur almost exclusively at the outer subcutaneous layer (OSQ) of the injection sites (Figure 1B,C). Meanwhile, H&E (hematoxylin and eosin) staining revealed the influx of inflammatory cells coinciding with the decrease in necrotic area, with fibrotic tissues appearing 1 week thereafter (Figure 1C,D). Based on these findings, s.c. injections of 1.5% MT921 and 1% DCA cause necrosis of subcutaneous fat, which are then cleared by inflammatory cells and replaced by fibrotic tissues, which leads to localized fat volume reduction. The complete panel of TTC- and H&E-stained tissue cross sections at different time points post-injection can be seen in Figures S1 and S2, respectively.

### 2.2. Effects of MT921 and DCA Subcutaneous Injections on Fat Layers and Skin of Mini Pigs

**Figure 2:**
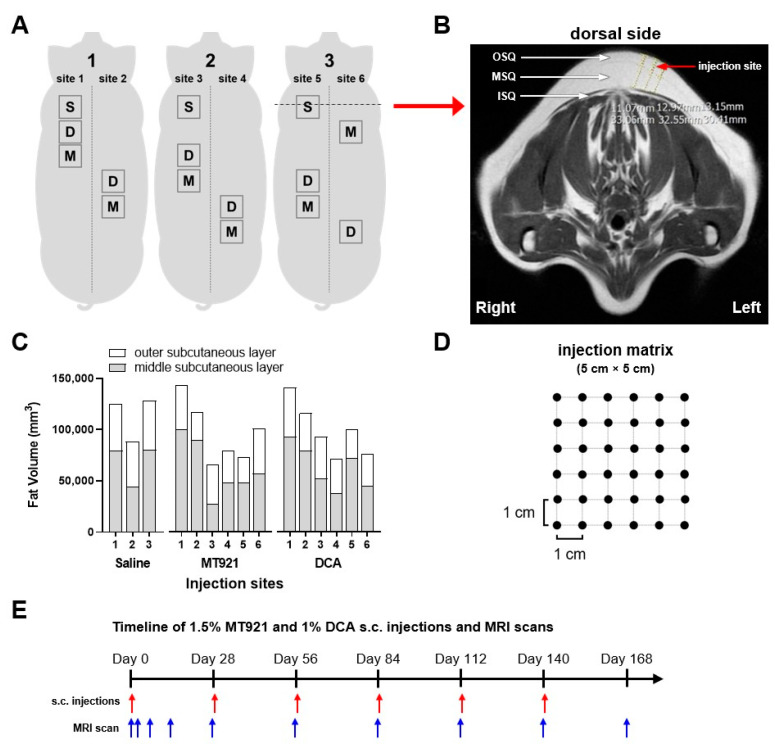
(**A**) Diagram illustrating **M**T921, **D**CA, and **S**aline injection sites at the back of mini pigs. (**B**) An MRI slice of a saline injection site showing the outer subcutaneous (OSQ), middle subcutaneous (MSQ), and inner subcutaneous (ISQ) layers; measurement lines mark the center and edges of the injection site. (**C**) Fat volumes of OSQ and MSQ at each injection site; data were analyzed using one-way ANOVA with Tukey’s multiple comparisons test. (**D**) Diagram of the injection matrix used for multiple s.c. injections at the injection sites. (**E**) Timeline of 1.5% MT921, 1% DCA, and saline repeat doses (0.2 mL, 5 mm depth) and MRI scans.

Localized fat volume reduction at the back of three mini pigs resulting from multiple s.c. injections of 1.5% MT921 and 1% DCA was evaluated and compared. Injection sites on the back of each mini pig, each having an area of 5 cm × 5 cm, were assigned to receive saline (1 site each), 1.5% MT921 (2 sites each), and 1% DCA (2 sites each). Fat volumes of the outer subcutaneous (OSQ) and middle subcutaneous (MSQ) layers at the injection sites were measured by magnetic resonance imaging (MRI) using T1-weighted transverse scans (Figure 2A,B). Volumetric analysis of the OSQ showed no significant difference in fat volumes among saline (45,658 ± 1875 mm^2^), MT921 (34,674 ± 8179 mm^2^), and DCA (36,159 ± 7202 mm^2^) injection sites. Similarly, MSQ fat volumes at saline (68,104 ± 20,839 mm^2^), MT921 (62,019 ± 27,693 mm^2^), and DCA (63,446 ± 21,560 mm^2^) injection sites showed no significant difference, though the degree of variability of higher compared to those of the OSQ (Figure 2C). A total of 36 s.c. injections of 1.5% MT921, 1% DCA, and saline (0.2 mL, 5 mm depth) was administered on each of their respective sites using an injection matrix, as shown in Figure 2D. Six repeated doses of the lipolytic substances were performed on the same sites at 4-week intervals, and MRI scans were conducted periodically to monitor changes in fat volume (Figure 2E).

#### 2.2.1. Effect of 1.5% MT921 and 1% DCA s.c. Injections on Outer Subcutaneous Layer

**Figure 3:**
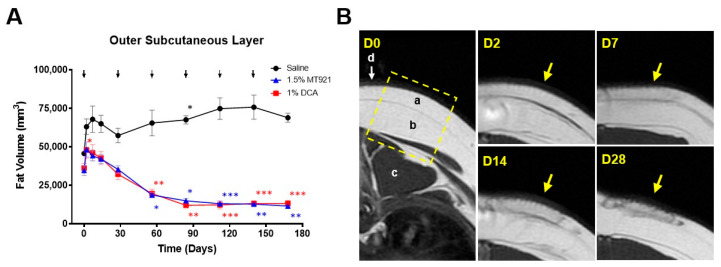
(**A**) Changes in OSQ fat volume at injection sites at the back of mini pigs following repeated s.c. doses of 1.5% MT921 (*n* = 6), 1% DCA (*n* = 6), and saline (*n* = 3); arrows indicate dose interval; data points and error bars represent mean ± standard error; asterisks denote significant difference in averge fat volumes (* *p* < 0.05, ** *p* < 0.01, *** *p* < 0.001) at MT921 (blue), DCA (red), and saline (black) injection sites from their initial values; data were analyzed using two-way ANOVA with Dunnett’s multiple comparisons test. Measurements were performed on day 0 (pre-injection); day 2, 7, 14, and 28; and every 4 weeks thereafter up to day 168 (24 weeks) after the first injection. (**B**) Representative MRI slices of an MT921 injection site at different time points after the first dose of 1.5% MT921; left-most panel: region of the injection site (dotted box), outer subcutaneous (a), middle subcutaneous (b), longgisimus muscle (c), dermis, and epidermis (d); four right panels: fat necrosis at the same injection site (yellow arrows) on day 2, 7, 14, and 28 post-injection.

After the first dose of 1.5% MT921, 1% DCA, and saline, a transient increase in OSQ fat volumes at their injection sites was observed in the first week and was likely caused by an inflammatory reaction to the invasive procedure. This reaction eventually subsided by day 28 and was no longer observed in subsequent doses. By day 140, OSQ fat volume at saline injection sites had gradually increased by an average of 30,066 mm^3^, or +65.44% of their initial volume, at the start of the study and could be attributed to the natural buildup of fat deposits [18]. Meanwhile, OSQ at injection sites administered with 1.5% MT921 and 1% DCA showed a significant decrease in fat volumes 4 weeks after the second dose (day 56), with an average reduction of 15,963 mm^3^ (−43.50%, *p* < 0.05) and 16,548 mm^3^ (−45.94%, *p* < 0.01), respectively. Four weeks after the third dose (Day 84), fat volumes at MT921 and DCA injection sites had decreased by an average of 19,798 mm^3^ (−54.83%, *p* < 0.05) and 24,164 mm^3^ (−66.26%, *p* < 0.01), respectively. By day 168, or 4 weeks after the sixth dose, fat volumes at MT921 and DCA injection sites had decreased by an average of 23,137 mm^3^ (−66.45%, *p* < 0.01) and 22,930 mm^3^ (−63.72%, *p* < 0.001), respectively. There was no significant difference in OSQ fat volume changes at MT921 and DCA injection sites throughout the course of treatment, indicating comparable fat-reducing effects between 1.5% MT921 and 1% DCA under the conditions of the study (Figure 3A, Table 1). OSQ fat volume reduction was closely associated with localized fat necrosis (Figure 3B).

#### 2.2.2. Effect of 1.5% MT921 and 1% DCA s.c. Injections on Middle Subcutaneous Layer

**Figure 4:**
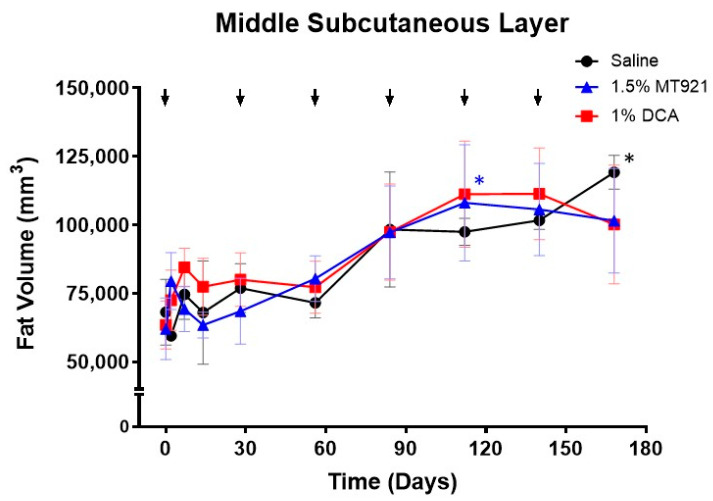
Changes in MSQ fat volume in mini pigs following repeated s.c. injections of 1.5% MT921 (*n* = 6), 1% DCA (*n* = 6), and saline (*n* = 3); arrows indicate dose interval; data points and error bars represent mean ± standard error; asterisks denote significant difference in averge fat volume (* *p* < 0.05) at MT921 (blue) and saline (black) injection sites from their initial values. Data were analyzed using two-way ANOVA with Dunnett’s multiple comparisons test. Measurements were performed up to day 168 (24 weeks) after the first injection.

Fat volumes of the MSQ at injection sites were also measured to determine whether repeated s.c. injections of 1.5% MT921 and 1% DCA would also result in MSQ fat volume reduction. Similar to the OSQ, transient increase in volume was observed in the MSQ 1 week after administering the first dose of saline, 1.5% MT921, and 1% DCA, albeit for a shorter period, as the reaction had subsided by day 14. In contrast to the OSQ, there was no significant decrease in MSQ fat volumes following subsequent doses of 1.5% MT921 and 1% DCA. On the contrary, fat volumes increased over time, comparable to those at saline injection sites up to day 140 (Figure 4, Table 2). By day 168, however, MSQ fat volume at saline injection sites had increased by an average of 51,100 mm^3^, or +85.36% of their initial volume (*p* < 0.05), while those at MT921 and DCA injection sites had only increased by an average of 39,492 mm^3^ (+66.04%) and 36,714 mm^3^ (+59.30%), respectively. Signs of MSQ fat reduction were observed at MT921 and DCA injection sites between day 140 and 168, 4 weeks after the fifth and sixth doses of 1.5% MT921 and 1% DCA, respectively. Since OSQ fat volume at MT921 and DCA injection sites has been shown to decrease beginning from day 56, it is speculated that subsequent s.c. injections of 1.5% MT921 and 1% DCA are likely delivered at a closer proximity to the MSQ. Diffusion of the lipolytic substances to the MSQ would therefore expose the underlying adipose tissue, resulting in fat necrosis and partial reduction in MSQ fat volume. Further investigation with additional repeated doses of the lipolytic substances would be required to establish this proposed mechanism.

#### 2.2.3. Skin Adverse Reactions Caused by 1.5% MT921 and 1% DCA s.c. Injections

**Figure 5:**
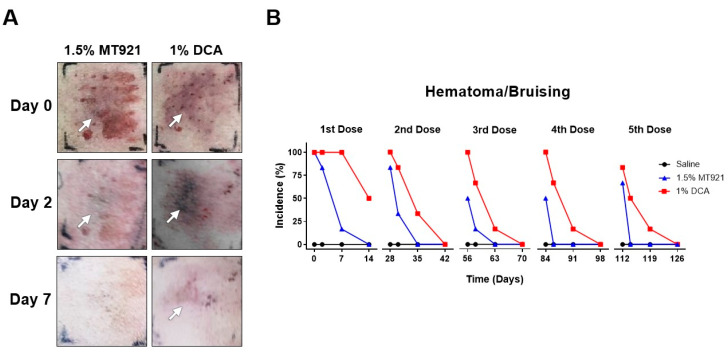
(**A**) Representative images of injection sites at the back of mini pigs showing hematoma (marked by arrows) following s.c. injections of 1.5% MT921 and 1% DCA. (**B**) Incidence rates and recovery time at among injection sites after each repeated dose of 1.5% MT921 (*n* = 6), 1% DCA (*n* = 6), and saline (*n* = 3). Incidence rates were compared using Student’s *t*-test; recovery times were analyzed using two-way ANOVA with Dunnett’s multiple comparisons test.

Aside from fat reduction, skin adverse reactions (ADR) resulting from repeated 1.5% MT921 and 1% DCA s.c. injections were also investigated. Hematoma (skin bruising) occurs during parenteral injections when the integrity of nearby blood vessels is compromised either through accidental puncture or due to the action of the drug being delivered, resulting in bleeding around the injection site. Here, hematoma was immediately observed in all six MT921 and DCA injection sites (100% incidence rate) after receiving the first dose. Drug action was considered as the cause since saline injection sites did not exhibit hematoma at any point in time during repeated s.c. injections of saline. Interestingly, subsequent doses of 1.5% MT921 to the same sites showed lower incidence of hematoma (second dose: 83.3%; third dose: 50%, 50%, and 66.7%) than 1% DCA (100%, 100%, 100%, and 83.3%) (*p* < 0.05). Moreover, at every dose of 1.5% MT921 (exception for the first dose), hematoma in most MT921 injection sites had faded after just 2 days (*p* < 0.05). By comparison, hematoma remained visible in most of the DCA injection sites 2 days after every dose of 1% DCA and only faded by 7 days post-injection (Figure 5).

**Figure 6:**
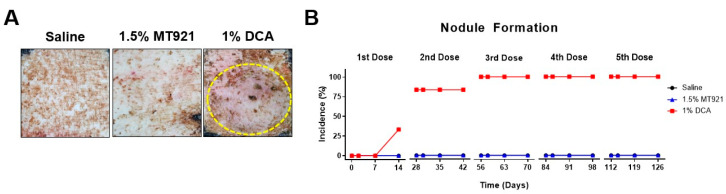
(**A**) Representative images of saline, MT921, and DCA injection sites at the back of mini pigs on day 56 (4 weeks after second s.c. dose of 1.5% MT921 and 1% DCA); nodule formation (marked by dotted circle) observed at the DCA injection site. (**B**) Incidence of nodule formation after each repeated s.c. dose of 1.5% MT921 (*n* = 6) and 1% DCA (*n* = 6), with saline as control (*n* = 3). Incidence rates were compared using one-way ANOVA.

Nodule formation was another SAE observed at injection sites caused by repeated multiple s.c. injections of the lipolytic substances, particularly 1% DCA (Figure 6A). As shown in Figure 6B, two of the six DCA injection sites developed nodules (33.3% incidence rate) 14 days after the first s.c. dose of 1% DCA. Before administering the second dose of 1% DCA on day 28, another three injection sites were found with similar nodules, raising the incidence rate to 83.3% (5 out of 6). By day 56, all six DCA injection sites (100%) had developed nodules, which persisted up to at least day 126. Similar nodules were not observed at MT921 and saline injection sites throughout the same period (*p* < 0.0001).

### 2.3. Ulcerative Dermatitis in ICR/CD1 Mice Caused by MT921 and DCA Subcutaneous Injection

Different concentrations of MT921 (0.5%, 1%, 1.5%, and 2%) and DCA (0.25%, 0.5%, 1%, and 1.5%) were each administered by s.c. injection (0.1 mL) to two sites on the backs of female ICR/CD1 mice (5 mice per group, total of 10 injection sites per test article) on day 0 and 2. Incidence and severity of ulcerative dermatitis (UD) on day 5 were graded using the dermatitis lesion scoring system (Table 3).

**Figure 7:**
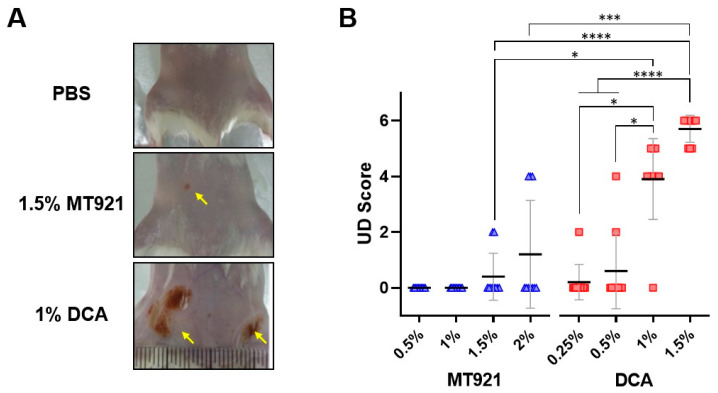
(**A**) Representative images showing ulcerative dermatitis (UD) at the two injection sites on the back of ICR/CD1 mice 5 days after s.c. injections of 1.5% MT921, 1% DCA, and PBS (total 0.2 mL per injection site); UD marked by arrows. (**B**) Dot plot of UD scores from each treatment (*n* = 10 injection sites each); horizontal line and error bars represent mean ± standard deviation; asterisks denote significant difference (* *p* < 0.05, *** *p* < 0.001, **** *p* < 0.0001). Data were analyzed using Kruskal–Wallis test.

As shown in Figure 7, s.c. injection of MT921 induced UD only at concentrations of 1.5% and 2%, whereas DCA induced UD at concentrations as low as 0.25%. Moreover, the two UD-positive 1.5% MT921-injected sites (20% incidence rate) and three UD-positive 2% MT921-injected sites (30% incidence rate) only registered a UD score of 2 and 4, respectively. By comparison, 9 of the UD-positive 1% DCA-injected sites (90% incidence rate) registered a UD score between 4 and 5, while all 10 UD-positive 1.5% DCA-injected sites (100% incidence rate) registered a UD score of at least 5. Taken together, MT921 s.c. injection elicited milder UD in mice than DCA, exemplified by the lower UD incidence and severity of 1.5% MT921 compared to 1% DCA (*p* < 0.05) and 1.5% DCA (*p* < 0.0001).

### 2.4. Local Edema in SD Rat Caused by MT921 and DCA Intraplantar Injection

**Figure 8:**
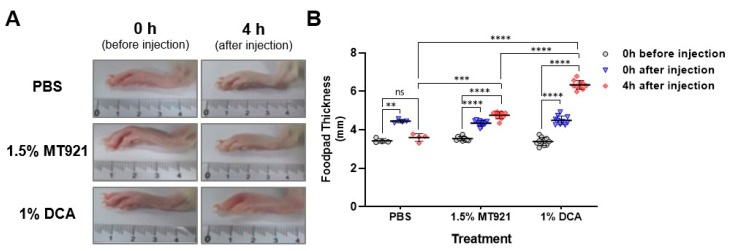
(**A**) Representative images showing development of edema on SD rat hind paws before and 4 h after intraplantar injections of 1.5% MT921, 1% DCA, and PBS (0.1 mL per injection). (**B**) Dot plot of footpad thickness for each treatment (*n* = 10 footpads each for MT921 and DCA, *n* = 4 footpads for PBS) at 0 h (before and after) and 4 h after administration; horizontal line and error bars represent mean ± standard deviation; asterisks denote significant difference (** *p* < 0.02, *** *p* < 0.001, **** *p* < 0.0001) and ns as not significant. Data were analyzed using two-way ANOVA with Dunnett’s and Tukey’s multiple comparisons tests.

Significant swelling was observed on the footpad of male SD rats immediately after intraplantar injections of 1.5% MT921 (*p* < 0.0001), 1% DCA (*p* < 0.0001), and PBS (*p* < 0.01), indicating that the immediate swelling is likely caused by acute reaction to the procedure or volume displacement at the injection sites by the injected solutions. The reaction was transient, as footpad thickness of PBS-injected hind paws returned to normal 4 h later. In contrast, footpads of 1.5% MT921- and 1% DCA-injected hind paws exhibited increased swelling 4 h post-injection (*p* < 0.0001), suggesting that the drug action of both lipolytic substances can elicit local edema. Interestingly, thicker footpads were observed in 1% DCA-injected hind paws compared to 1.5% MT921-injected hind paws 4 h post-injection (*p* < 0.0001), indicating 1.5% MT921 causes milder edema on the hind paws of SD rats compared to 1% DCA (Figure 8).

## 3. Discussion

In injection lipolysis, bile acid salts such as sodium deoxycholate and sodium cholate act as amphiphilic surfactants capable of disrupting adipocyte membranes through micelle-mediated solubilization of lipids and cholesterol. Injectable formulation of both compounds was prepared by dissolving their bile acid forms (DCA and CA, respectively) in phosphate buffer containing sodium chloride and proportionate amount of sodium hydroxide to generate their salt (anionic) forms, which exhibit detergent properties under physiological conditions (final pH ≈ 8 for DCA and ≈7 for CA). Above their critical micelle concentrations, these anionic surfactants incorporate into the phospholipid bilayer and form mixed micelles with membrane lipids and cholesterol, leading to dissolution of membrane components, loss of osmotic stability, and eventual cell lysis [2,7,19]. Exposure of adipocytes to these bile acids can lead to cell damage, apoptosis, and leakage of cellular contents, including fat deposits. Alternatively, these bile acids may also indirectly cause fat necrosis by inducing inflammation [19]. Studies on ATX-101 have shown that exposure of subcutaneous fat tissue to 1% DCA causes the destruction of adipocyte cell membranes and release of fat deposits that are eventually cleared by the lymphatic system. The overall number of adipocytes and fat deposits at the area targeted by DCA decreases and, as a consequence, reduces the volume of the subcutaneous tissue [20].

In the present study, we demonstrated the similarity in the sequence of histopathological events caused by subcutaneous (s.c.) injection of 1.5% MT921 and 1% DCA. Both articles induced comparable localized necrosis of adipose tissue, accompanied by the influx of inflammatory cells at the affected area that clears the fat deposits and cellular debris, followed by the formation of fibrotic tissue. Not surprisingly, repeated multiple s.c. injections of 1.5% MT921 and 1% DCA also resulted in comparable reduction of fat volume, particularly at the outer subcutaneous layers since it was anticipated that the depth of injections on the back of the mini pigs would deliver these lipolytic substances to this region of the skin.

Although injection lipolysis with DCA and MT921 is considered minimally invasive, the procedure still involves introducing a bile acid into the hypodermis, which would normally trigger skin adverse events. DCA injections have been reported to cause mild-to-moderate skin reactions, such as bruising (hematoma), swelling (edema and nodule formation), pain, and itching at the injection sites. In some serious cases, allergic reactions, skin ulcers, and permanent scarring have also been reported [10,11]. Meanwhile, clinical study on MT921 has also proven to be safe, as well as highly tolerable, since pain and edema at the injection sites were the only notable adverse events, and these eventually resolve over time [13].

In our mini pig model, both 1.5% MT921 and 1% DCA caused bruising at the injection sites with the initial treatment. However, subsequent injections of 1.5% MT921 recorded lower incidence rates compared to 1% DCA. In addition, bruising at the sites injected with 1.5% MT921 resolved faster than those injected with 1% DCA. Moreover, none of the sites injected with 1.5% MT921 developed nodules throughout the course of treatment, whereas all sites injected with 1% DCA had formed nodules after the second dose. Our skin adverse reaction studies using rodent models had also shown that, compared to DCA, MT921 had caused milder case of skin ulcers and foot swelling in mice and rats, respectively. These observations suggest that while their pharmacologic properties are highly similar, the biochemical properties of MT921 confer milder skin reactions compared to that of DCA through a mechanism that is yet to be identified.

Multiple factors influence injection-site pain. These include injection location, injected volume, injection rate, and, more importantly, the pH of the formulation. Solutions outside the range of physiologic pH are known to provoke tissue irritation, leading to increased discomfort or pain at the injection site [21,22]. For bile acids like DCA and CA, solubility is strongly pH-dependent. Patent data for MT921 indicate that CA begins to precipitate below pH 6.7, while DCA precipitates or forms gels below pH 8.0 [23]. Supporting evidence for DCA formulations shows that aqueous solutions of DCA salts at concentrations of approximately 0.4–2% *w*/*v* require buffering at pH 8.1–8.5 to prevent precipitation and maintain solubility [24]. As a consequence, the relatively alkaline conditions required for DCA formulations may contribute to greater risk of tissue irritation and pain upon injection. In contrast, CA can be formulated more stably at physiological pH. This would imply that the physicochemical differences may provide CA-based formulations a potential advantage over DCA-based drugs in reducing skin adverse reactions at the injection sites.

The present study demonstrated the comparability of fat-reducing efficacy between 1.5% MT921 and 1% DCA subcutaneous injections in the mini pig model. Furthermore, MT921 was shown to elicit milder skin adverse reactions compared to DCA. Based on these observations, along with the observations from the clinical studies, MT921 is a promising alternative to DCA-based drugs for injection lipolysis.

## 4. Materials and Methods

### 4.1. Animals

Four SPF (specific pathogen-free) female mini pigs (MICROPIG, 42.3–47.5 kg, 21–31 months old) were purchased from Apures Co., Ltd. (formerly MediKinetics, Seoul, Republic of Korea) and housed at the animal facility of KNOTUS Co., Ltd. (Incheon, Republic of Korea). Mini pigs were selected for investigating subcutaneous fat reduction efficacy since their skin architecture and subcutaneous fat composition closely resemble those of humans, making them a suitable translational model for evaluating injection lipolysis. A total of 42 6-week-old female ICR/CD1 mice from Orient Bio, Inc. (Seoul, Republic of Korea) and 12 6-week-old male Sprague–Dawley (SD) rats from Koatech Co., Ltd. (Seoul, Republic of Korea) were housed at the animal facility of Medytox Gwanggyo R&D Center (Medytox Inc., Seoul, Republic of Korea). Animals were kept in a highly controlled environment (23 ± 3 °C, 15% relative humidity, 12 h light/dark cycle, 10–20 air changes per hour) and given free access to food and water. A 1-week quarantine and acclimation period was imposed on all animals prior to the start of the experiments. At the conclusion of the study, all remaining animals were euthanized either by overdose of anesthesia (mini pigs) or CO_2_ asphyxiation (mice and rats). All procedures involving laboratory rodents and mini pigs were approved by the Institutional Animal Care and Use Committees (IACUC) of Medytox Inc. (A-2014-006 and A-2014-015) and KNOTUS Co., Ltd. (18-KE-094-1), respectively, and in accordance with the Laboratory Animal Act of Korea (Act No. 15278).

### 4.2. Test Articles

In mini pig studies, 1% (*w*/*v*) DCA solution was prepared with the same composition as Kybella^®^ (without the preservative), consisting of 10 mg/mL deoxycholic acid (CAS No. 83-44-3, ≥98% purity, Sigma, St. Louis, MO, USA) as active ingredient and 2.68 mg/mL Na_2_HPO_4_·7H_2_O (Cat# S9390, Lot# SLBQ2859V, Sigma-Aldrich), 4.38 mg/mL NaCl (Cat# 106404, Lot# K48327304643, Merck, Darmstadt, Germany), and 1.43 mg/mL NaOH (Cat# 106469, Lot# B1278669633, Merck) as excipients. The final solution was adjusted to pH 8.3 using HCl (Cat# 1.00314, Merck). Meanwhile, 1.5% MT921 solution was prepared with the same composition as the MT921 clinical trial batch [13] and contains 15 mg/mL cholic acid (CAS No. 81-25-4, ≥98% purity, Prodotti Chimici e Alimentari S.P.A., Basaluzzo, Italy) as active ingredient and excipients comprised of sodium phosphate buffer, sodium chloride, and sodium hydroxide. Preservative-free normal saline (Lot# H7R8AF3, Dai Han Pharm Co., Ltd., Seoul, Republic of Korea) was used as control. In rodent studies, 0.25~1.5% DCA (pH8.3) and 0.5~2% MT921 (pH7.0) solutions were prepared by dissolving sodium deoxycholate (CAS No. 302-95-4, ≥97% purity, Sigma-Aldrich) and sodium cholate (CAS No. 361-09-1, ≥96% purity, CalbioChem, San Diego, CA, USA) in sodium phosphate buffer with NaCl. Phosphate-buffered saline (PBS) (Cat.# LB004-02, WelGene, Gyeongsan, Republic of Korea) was used as control.

### 4.3. Experimental Design Summary

A summary of the experimental design for each animal study, including animal species, sample size, dosing regimen, and key assessments, is provided in Table 4.

### 4.4. MT921 and DCA Subcutaneous Injections in Mini Pigs

One mini pig was used to investigate the sequence of histopathological events. Three mini pigs were used to evaluate volume reduction in the outer subcutaneous (OSQ) and middle subcutaneous (MSQ) and skin adverse events. Prior to article administration, MRI scan, and tissue excision, parenteral anesthesia was administered using a single intramuscular (i.m.) injection of 5 mg/kg Zoletil^®^ 50 (VIRBAC, Carros, France) and 2.5 mg/kg xylazine (Rompun^®^, Bayer AG, Leverkusen, Germany), supplemented with respiratory anesthesia using isoflurane (Hana Pharm Co., Ltd., Seoul, Republic of Korea) when necessitated. Dorsal regions were shaved, and injection sites were labeled using permanent marker.

#### 4.4.1. Histopathological Examinations

Sequence of histopathological events was investigated using a reverse-time course approach. Single subcutaneous (s.c.) injections of 1.5% MT921 and 1% DCA (0.2 mL, 5 mm depth) were each administered to two adjacent sites on the back of a mini pig at 7-day intervals for up to 56 days, as shown in Figure 1A. One h after the last injection, tissue samples from all injection sites and four non-injected sites were excised, washed with PBS, and sectioned in halves along the sagittal plane across the center of the injection site. One half of the tissue cross sections were incubated in 2% TTC solution (2,3,5,-Triphenyl-tetrazolium chloride; Cat.# T8877, Lot# BCBV1635, Sigma) for 16 to 18 h at room temperature in the dark, while the other halves were fixed using 10% formalin (Cat.# HT501320, Lot# SLBR9444V, Sigma) for 3 to 4 days at room temperature and embedded in paraffin. Digital images of TTC-stained tissue cross sections were acquired using Epson Perfection V700 Photo Scanner (Epson, Los Alamitos, CA, USA) and analyzed using ImageJ v1.52 (NIH, Bethesda, MD, USA) [25]. Area of necrotic tissue, which appears as unstained regions against a red background caused by the enzymatic reduction of TTC to red formazan compound, was calculated using the free hand selection tool. Meanwhile, 5 μm thin sections of the paraffin-embedded tissues prepared using a Microm HM 340E microtome (Thermo Fisher Scientific, Waltham, MA, USA) were mounted onto glass slides and stained with hematoxylin (Cat.# HHS32, Lot# SLBP6178V, Sigma-Aldrich) and eosin (Cat.# C0773, Lot# MKB28859V, Diapath, Bergamo, Italy) and then observed using Leica DMi1 stereo microscope (Leica, Tokyo, Japan). Inflammatory cell influx and fibrosis observed from H&E-stained sections were each graded using a 4-point scale as follows: 1 (mild), 2 (mild-to-moderate), 3 (moderate), or 4 (severe) based on criteria described elsewhere [26].

#### 4.4.2. Changes in Volume of Subcutaneous Fat Layers

Five injection sites on the back of three mini pigs received six repeated doses of 1.5% MT921 (2 sites each), 1% DCA (2 sites each), and saline (1 site each) at 4-week intervals according to the layout shown in Figure 2A. The 4-week dosing interval schedule was based on histopathological events observed from the single-dose mini pig study and was consistent with clinical injection schedules reported for deoxycholic acid-based injection lipolysis treatments [11]. Each dose was comprised of 36 s.c. injections (0.2 mL, 5 mm depth) spaced at 1 cm interval in a 5 cm × 5 cm grid (see Figure 2D) for a volume-adjusted dose of 0.2 mL/cm^3^ saline, 3 mg/cm^3^ MT921, and 2 mg/cm^3^ DCA. T1-weighted transverse MRI scans of the injection sites, demarcated by titanium wires during imaging, were acquired on day 0 (prior to the initial dose), 2, 7, 14, and 28 and every 4 weeks thereafter up to day 168 using a MAGNETOM Essenza 1.5T magnetic resonance imaging (MRI) system (Siemens Co., Munich, Germany). Volumetric measurement of the OSQ and MSQ at the injection sites was performed using ImageJ following the method described elsewhere [27,28].

#### 4.4.3. Skin Adverse Reactions

For the first five repeated dose of the test articles, injection sites were visually inspected for local skin adverse reactions (ADR) immediately after each dose 2, 7, and 14 days thereafter. Incidence rates of hematoma and formation of nodules based on the proportion of injection sites positive for the ADR were recorded.

### 4.5. Ulcerative Dermatitis in Mice

Prior to administration of the test articles, ICR/CD1 mice were anesthetized with an intraperitoneal (i.p.) injection of 60 mg/kg ketamine (Yuhan Corp., Seoul, Republic of Korea) and 11.5 mg/kg xylazine. After shaving the lower back of the animals, four different concentrations of MT921 (0.5%, 1%, 1.5%, and 2%) and DCA (0.25%, 0.5%, 1%, and 1.5%) were each administered to groups of five mice. Each mouse received two 0.1 mL s.c. injections of the test articles on opposite side of the dorsal midline on day 0 and 2. As a control, two other mice were administered PBS in the same manner. On day 5, all animals were euthanized and inspected for dermatitis lesions at the injection sites. Severity of the lesions at each injection site was scored from 0 to 6 based on the character and size of the lesions following the ulcerative dermatitis (UD) criteria described elsewhere [29], with some modifications (see Table 3).

### 4.6. Edema in Rat Footpads

Prior to administration of the test articles, SD rats were anesthetized with an i.p. injection of 60 mg/kg ketamine and 12.5 mg/kg xylazine. Two groups of five SD rats were each given 0.1 mL intraplantar injections of 1.5% MT921 or 1% DCA to both hind paws. For the control, two SD rats were administered PBS in the same manner. Footpad thickness at 0 h (before and after) and 4 h after administration of the articles was measured using a Bluebird digital caliper (Misumi, Schaumburg, IL, USA).

### 4.7. Statistical Analysis

Data are expressed as mean ± standard deviation (SD), frequency, or percentages, where indicated. For comparative analysis, one- or two-way analysis of variance (ANOVA) with Dunnett’s or Tukey’s multiple comparisons test, two-tailed Student’s *t*-test, or Kruskal–Wallis test was applied. A *p*-value less than 0.05 was considered statistically significant. Statistical analyses were performed using GraphPad Prism 7.05 (GraphPad Software, San Diego, CA, USA).

## 5. Patents

Parts of this study, including the findings on skin adverse events in mice and foot edema in rats, are covered by a registered patent (Patent No. 10-2146667, Intellectual Property Office of the Republic of Korea).

## Figures and Tables

**Table 1:** Changes in OSQ fat volume at saline, MT921, and DCA injection sites.

Time	Fat Volume (mm^3^)			Volume Increase (+) or Decrease (−) Since Day 0 (mm^3^)		
(Days)	Saline (*n* = 3)	1.5% MT921 (*n* = 6)	1% DCA (*n* = 6)	Saline (*n* = 3)	1.5% MT921 (*n* = 6)	1% DCA (*n* = 6)
0	45,658 ± 1875	34,674 ± 8179	36,159 ± 7202	n/a	n/a	n/a
2	63,008 ± 8845	48,257 ± 11,777	47,946 ± 12,815 *	(+) 17,351 ± 8618	(+) 13,583 ± 9613	(+) 11,787 ± 6325
7	67,912 ± 14,876	44,165 ± 7861	46,213 ± 13,069	(+) 22,255 ± 16,137	(+) 9491 ± 6460	(+) 10,054 ± 7401
14	65,004 ± 9573	42,102 ± 7347	42,762 ± 10,585	(+) 19,346 ± 9870	(+) 7427 ± 5342	(+) 6603 ± 5356
28	57,327 ± 8084	35,363 ± 6027	32,181 ± 8607	(−) 11,669 ± 7279	(−) 688 ± 5857	(−) 3978 ± 5825
56	65,453 ± 14,410	18,712 ± 4332 *	19,611 ± 6747 **	(+) 19,795 ± 14,751	(−) 15,963 ± 8915	(−) 16,548 ± 5219
84	67,541 ± 4743 *	14,876 ± 3984 *	11,995 ± 2738 **	(+) 21,884 ± 4548 ^a^	(−) 19,798 ± 9167 ^b^	(−) 24,164 ± 6440 ^b^
112	74,846 ± 12,128	13,054 ± 3895 ***	12,275 ± 6427 ***	(+) 29,188 ± 12,669 ^a^	(−) 21,611 ± 4774 ^b^	(−) 23,884 ± 4208 ^b^
140	75,724 ± 13,533	12,768 ± 2643 **	13,224 ± 3853 ***	(+) 30,066 ± 12,230 ^a^	(−) 21,906 ± 7271 ^b^	(−) 22,935 ± 5671 ^b^
168	68,909 ± 5027	11,537 ± 3655 **	13,229 ± 4575 ***	(+) 23,251 ± 6900 ^a^	(−) 23,137 ± 6321 ^b^	(−) 22,930 ± 4775 ^b^

OSQ, outer subcutaneous layer; MT921, cholic acid; DCA, deoxycholic acid; n/a, not applicable. Data shown as mean ± SD. Asterisks denote significant difference (* *p* < 0.05, ** *p* < 0.01, *** *p* < 0.001) compared to day 0; annotations that do not share a common superscript (^a, b^) denote significant difference between treatment groups (*p* < 0.05 or less) at the given time point. Data were analyzed using two-way ANOVA with Dunnett’s (for fat volume) or Tukey’s (for +/− in fat volume) multiple comparisons tests.

**Table 2:** Changes in MSQ fat volume at saline, MT921, and DCA injection sites.

Time	Fat Volume (mm^3^)			Volume Increase (+) or Decrease (−) Since Day 0 (mm^3^)		
(Days)	Saline (*n* = 3)	1.5% MT921 (*n* = 6)	1% DCA (*n* = 6)	Saline (*n* = 3)	1.5% MT921 (*n* = 6)	1% DCA (*n* = 6)
0	68,104 ± 20,839	62,019 ± 27,693	63,446 ± 21,560	n/a	n/a	n/a
2	59,510 ± 1602	79,461 ± 25,586	72,533 ± 27,083	(−) 8594 ± 22,440	(+) 17,442 ± 15,147	(+) 9087 ± 18,399
7	74,604 ± 15,779	69,247 ± 20,122	84,506 ± 16,983	(+) 6501 ± 22,544	(+) 7229 ± 14,811	(+) 21,060 ± 14,927
14	67,971 ± 32,740	63,413 ± 11,525	77,391 ± 25,529	(−) 133 ± 29,357	(+) 1394 ± 23,590	(+) 13,945 ± 14,355
28	76,888 ± 15,458	68,417 ± 29,421	79,950 ± 23,994	(+) 8783 ± 11,796	(+) 6399 ± 16,658	(+) 16,504 ± 19,175
56	71,516 ± 9529	80,395 ± 20,362	77,239 ± 23,358	(+) 3412 ± 25,412	(+) 18,376 ± 18,026	(+) 13,793 ± 18,630
84	98,288 ± 36,324	97,289 ± 41,537	97,396 ± 43,013	(+) 30,184 ± 52,311	(+) 35,270 ± 21,922	(+) 33,950 ± 38,760
112	97,430 ± 8497	108,089 ± 51,954 *	111,236 ± 47,491	(+) 29,326 ± 15,096	(+) 46,070 ± 28,264	(+) 47,790 ± 36,261
140	101,668 ± 5697	105,636 ± 41,212	111,344 ± 41,072	(+) 33,564 ± 15,241	(+) 43,617 ± 27,991	(+) 47,898 ± 35,035
168	119,204 ± 10,666 *	101,510 ± 46,670	100,160 ± 53,117	(+) 51,100 ± 10,994	(+) 39,492 ± 25,877	(+) 36,714 ± 42,871

MSQ, middle subcutaneous layer; MT921, cholic acid; DCA, deoxycholic acid; n/a, not applicable. Data shown as mean ± SD. Asterisks denote significant difference (* *p* < 0.05) compared to day 0; comparison between groups found no statistically significant difference at any time point. Data was analyzed using two-way ANOVA with Dunnett’s (for fat volume) or Tukey’s (for +/− in fat volume) multiple comparisons tests.

**Table 3:** Scoring system for ulcerative dermatitis (UD) lesion in mice ^1^.

Character of Lesion	Score	Length of Lesion	Score
No lesion	0	0 cm	0
One, small punctuate crust	1	<0.2 cm	1
Multiple, small punctuate or coalescing crust	2	0.2~0.5 cm	2
Erosion or ulceration	3	>0.5 cm	3

^1^ UD score is the sum of scores for “Character of Lesion” and “Length of Lesion”.

**Table 4:** Summary of Experimental Design.

Model	No. of Animals	Treatment Groups	Dosing Regimen	Main Endpoints	Study Duration
Mini pig (MICROPIG)	1	1.5% MT921, 1% DCA	Single s.c. injection at multiple dorsal sites	Sequential histopathology (necrosis, fibrosis, inflammation)	Day 1–56
Mini pig (MICROPIG)	3	1.5% MT921, 1% DCA	36 s.c. injections (5 cm × 5 cm matrix at multiple dorsal sites) every 4 weeks × 6 times	MRI fat volume change, local ADRs	Up to day 168
Mouse (ICR/CD1)	5/group	MT921 (0.5%, 1%, 1.5%, and 2%), DCA (0.25%, 0.5%, 1%, and 1.5%)	Single s.c. injection at left and right dorsal sites on day 0 and 2	Ulcerative dermatitis scoring	Day 5
Rat (Sprague–Dawley)	5/group	1.5% MT921, 1.0% DCA	Single intraplantar injection on both hind paws	Footpad edema (thickness)	4 h

s.c., subcutaneous; ADRs, adverse reactions; MRI; magnetic resonance imaging.

## Data Availability

Raw data supporting the conclusions of this article are proprietary and will be made available by the corresponding author on request.

## Supplementary Materials

The following supporting information can be downloaded at: https://www.mdpi.com/article/10.3390/ph18111643/s1, Figure S1: TTC-stained tissue cross section of MT921 and DCA injection sites at the back of a mini pig at different time points post-injection. Figure S2: H&E-stained tissue cross sections of MT921 and DCA injection sites at the back of a mini pig at different time points post-injection.

## Abbreviations

The following abbreviations are used in this manuscript:

CA	cholic acid
DCA	deoxycholic acid
MRI	magnetic resonance imaging
MSQ	middle subcutaneous layer
OSQ	outer subcutaneous layer
UD	ulcerative dermatitis
