# Supplementary material for: Comparison of Cholic Acid (MT921) and Deoxycholic Acid (DCA) in Fat Reduction Efficacy and Skin Adverse Reactions in Mini Pigs and Rodent Models
Source: Pharmaceuticals (Basel). 2025 Oct 30;18(11):1643. doi: 10.3390/ph18111643 (PMC12655580; doi:10.3390/ph18111643)
Supplement: Supplementary file 1 [file pharmaceuticals-18-01643-s001.zip › pharmaceuticals-3932115-supplementary.pdf]

Supplementary Materials

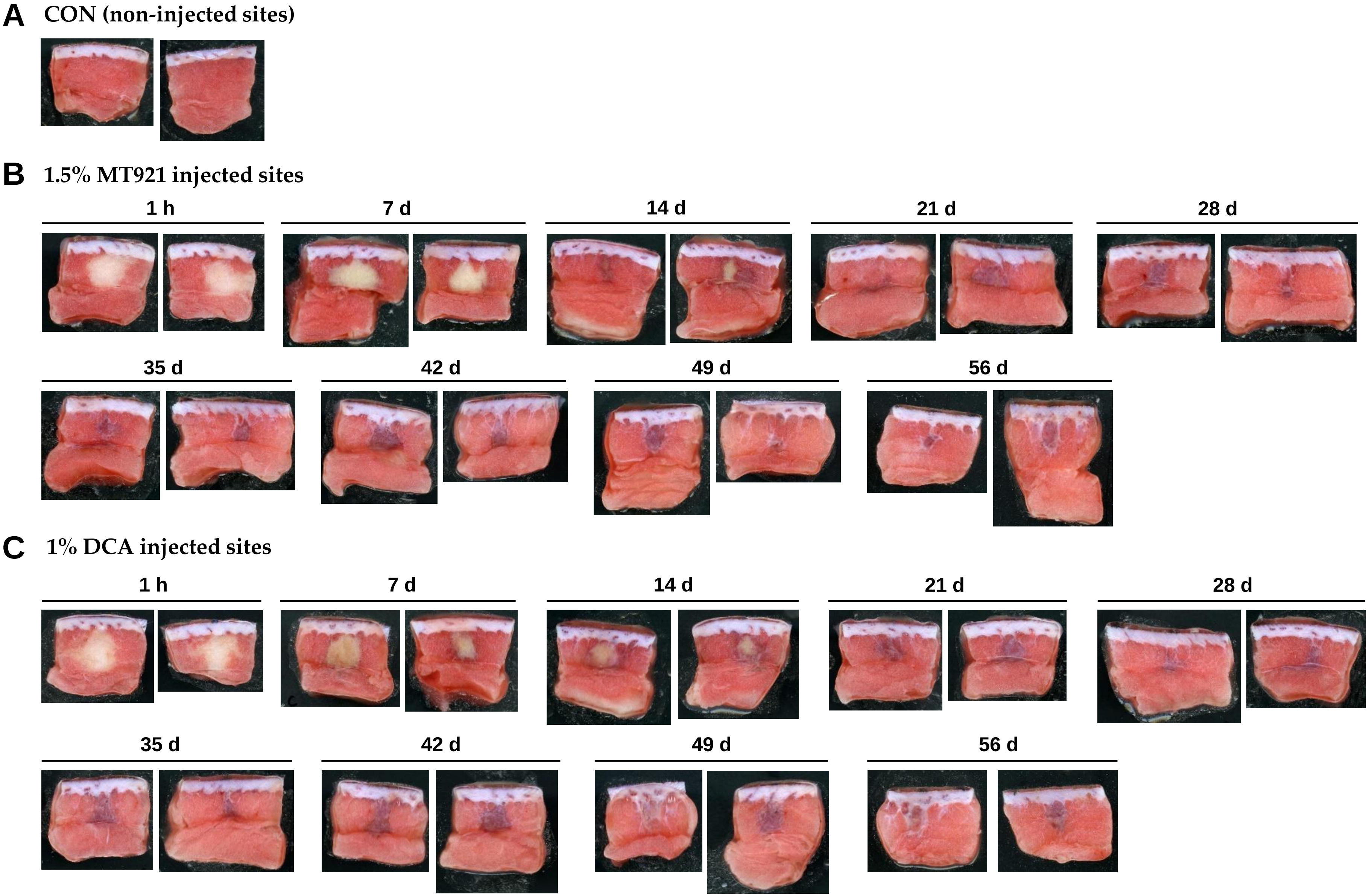

Figure S1. TTC-stained tissue cross section of MT921 and DCA injection sites at the back of a mini pig at different time points post-injection. (**A**) non-injected sites, (**B**) sites administered with a single s.c. dose of 0.2 mL 1.5% MT921, and (**C**) injection sites administered with a single s.c. dose of 0.2 mL 1% DCA.

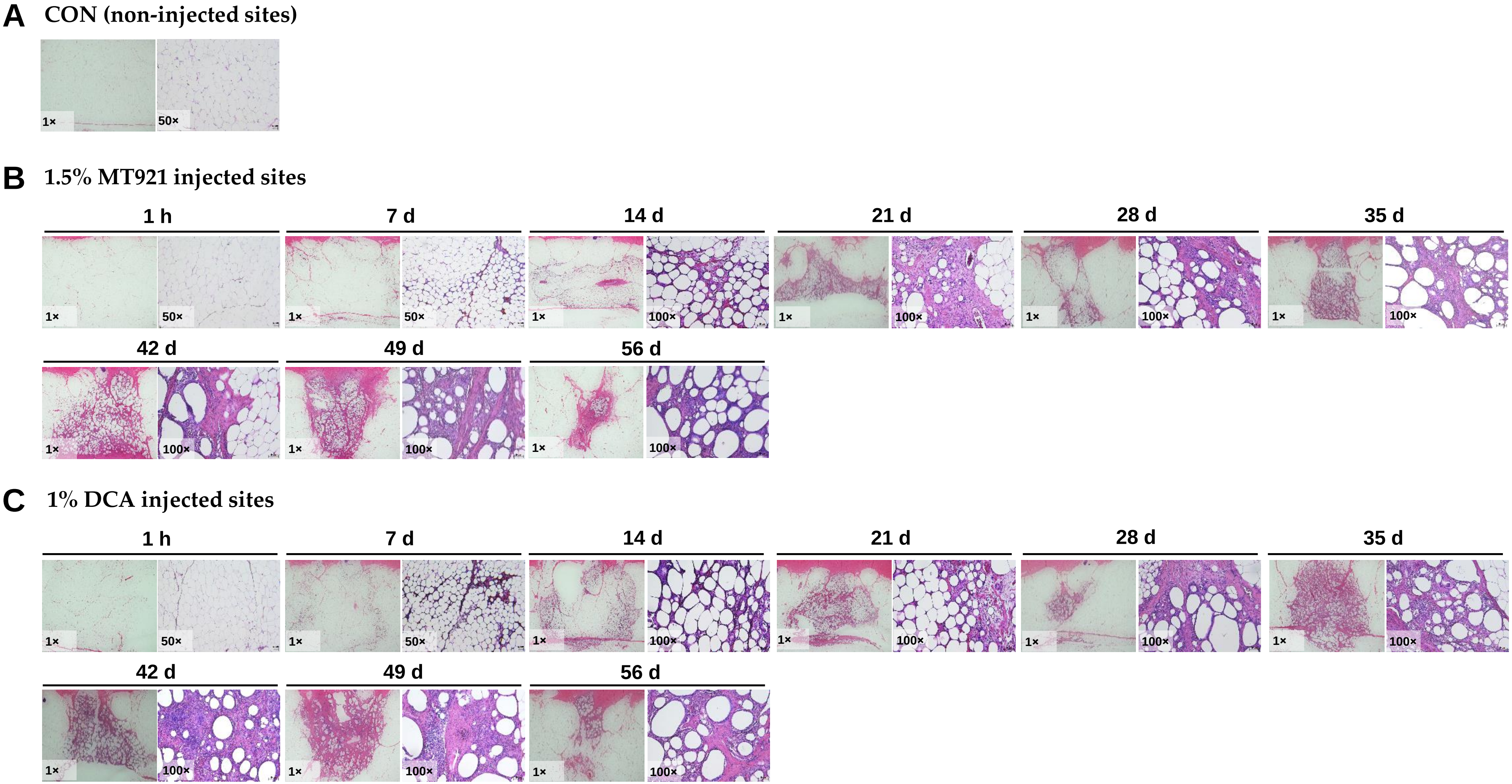

Figure S2. Hematoxylin & eosin (H&E)-stained tissue cross section of MT921 and DCA injection sites at the back of a mini pig at different time points post-injection. Representative images of a (**A**) non-injected site, (**B**) sites administered with a single s.c. dose of 0.2 mL 1.5% MT921, and (**C**) sites administered with a single s.c. dose of 0.2 mL 1% DCA. Digital images acquired using a stereomicroscope are shown at 1×; and 50× or 100× magnification.
